# Editorial: Phytonutrients in the treatment of cancer: Mechanistic insights and therapeutic prospects

**DOI:** 10.3389/fnut.2023.1134716

**Published:** 2023-03-07

**Authors:** Haroon Khan, Thomas Efferth, Silvia Tejada

**Affiliations:** ^1^Department of Pharmacy, Abdul Wali Khan University, Mardan, Pakistan; ^2^Department of Pharmaceutical Biology, Institute of Pharmaceutical and Biomedical Sciences, Johannes Gutenberg University, Mainz, Germany; ^3^Laboratory of Neurophysiology, Department of Biology, University of Balearic Islands, and IdISBa and CIBEROBN, Palma, Spain

**Keywords:** phytonutrients, cancers, preclinical studies, mechanistic insights, clinical status, drug discovery

Cancer is probably one of the leading causes of death worldwide, irrespective of age, gender, and locality. Recent statistic from 183 countries revealed that cancer represents the first or second cause of mortality in 112 countries of the world and the third or fourth cause in another 23 countries. Indeed, it is a huge economic burden affecting both individuals and health care systems (1). No doubt, cancer treatment is always a great challenge and therapeutic options are limited (2). The therapeutic approaches include surgery, chemotherapy, radiotherapy, and more recently immunotherapy depending upon the individual conditions with large variations in treatment success from case to case.

From a clinical perspective, many chemotherapeutic agents are non-sufficient in terms of sensitivity, efficacy, and safety and therefore shaken the confidence of prescribers. Natural products have served as the foundation for numerous therapeutic agents over the years, as they are more acceptable due to their organic nature, fair trade, and sustainably produced. Natural products consumption has significantly increased in recent years, resulting in the emergence of natural brands and specialty stores that focus solely on these products (3). Natural products have distinct characteristics that distinguish them from conventional synthetic molecules, providing both advantages and challenges for the drug discovery process. An increased awareness of ingredients in various products, accompanied by firm opinions about which ingredients or characteristics may or may not harm consumers, is driving these desires. It has been discussed that several components in natural products could have a significant anticancer potential that must be scientifically proven before new chemotherapeutics can be developed (4, 5). Quite a large number of newly isolated compounds are under preclinical and clinical investigation. In this context, the development of new techniques has greatly facilitated drug discovery and development from natural agents for cancer treatment.

In this Research Topic, we focus on secondary metabolites from natural food sources with strong effects as anticancer agents in both preclinical and clinical phases. Evidence-based studies on natural products with detailed mechanistic analyses are presented that could be candidates of clinical studies.

## Author contributions

All authors listed have made a substantial, direct, and intellectual contribution to the work and approved it for publication.
